# Microlearning in medical histology: a longitudinal evaluation of faculty-created five-minute videos using five theoretical frameworks and Kirkpatrick assessment

**DOI:** 10.1186/s12909-026-09900-6

**Published:** 2026-07-14

**Authors:** Anikó Szabó

**Affiliations:** Department of Anatomy and Genetics, College of Medicine, Alfaisal University, Riyadh, Saudi Arabia

**Keywords:** Microlearning, Medical histology education, Kirkpatrick evaluation, Faculty-created five-minute videos, Generation Z, Cognitive load theory, Multimedia learning theory, Variation theory, Spaced repetition theory, Self-regulated learning theory

## Abstract

**Background:**

Medical histology education worldwide faces a documented crisis: curriculum compression has dramatically reduced laboratory instructional time while content expectations remain unchanged. Microlearning, in the form of faculty-created five-minute histology review videos, offers a pragmatic and evidence-based contribution, but empirical evidence for its value in tissue identification remains limited. This study provides rigorous longitudinal evidence across three Kirkpatrick levels to address this gap.

**Methods:**

This longitudinal study followed first-year medical students across three time points within one semester at Alfaisal University, Saudi Arabia. Performance was assessed using a mock-OSPE on blood vessel histology. Students had access to 11 faculty-created five-minute histology review videos covering cardiovascular and renal histology.

Four hypotheses were tested across Kirkpatrick levels 1-3, guided by five theoretical frameworks: Cognitive Load Theory, Multimedia Learning Theory, Variation Theory, Spaced Repetition Theory, and Self-Regulated Learning Theory. Two hypotheses addressed Kirkpatrick Level 3 behavior. Two frameworks — Variation Theory and Self-Regulated Learning Theory — were applied to histology video education for the first time, to the best of the author's knowledge.

**Results:**

Mock-OSPE scores were significantly higher in the post-intervention assessment, with a large effect size. The majority of students watched the videos multiple times across both organ systems. Positive perceptions remained consistently high across all three time points, while negative perceptions remained low overall. Faculty-created videos exhibited a 17-fold adoption increase, becoming students' primary out-of-class learning method. Students valued both videos and face-to-face microscopy, consistent with Generation Z's blended learning profile.

**Conclusions:**

Faculty-created five-minute histology review videos are an effective and reproducible microlearning component of a broader strategy to mitigate the effects of curriculum compression. They extended learning beyond limited laboratory hours through 24/7 access, were associated with higher tissue identification scores, and showed high voluntary adoption — complementing rather than replacing hands-on learning.

**Supplementary Information:**

The online version contains supplementary material available at 10.1186/s12909-026-09900-6.

## Background

Medical schools face significant time constraints that threaten educational quality and demand new approaches from educators [1, 2]. The recent combination of visual learning and technological innovation offers a pragmatic response to these challenges [3]. Digital breakthroughs now enable anyone to create videos that previously required professional film production expertise [3]. Consequently, a single faculty member at Alfaisal University College of Medicine developed and evaluated a collection of five-minute histology review videos as a microlearning intervention.

### Global crisis in medical histology education

Medical histology education worldwide faces a documented crisis: curriculum compression has dramatically reduced histology laboratory instructional time while content expectations remain unchanged or have increased [1, 2, 4–7].

This global decline encompasses all four anatomical sciences: gross anatomy, histology, neuroanatomy, and embryology. Educators worldwide navigate similar pressures of reduced instructional time and increased curricular integration, with some suggesting that instruction has fallen below safe levels for clinical practice [2, 8].

Microscopic education develops visual analytical thinking. When laboratories disappear, this capacity is reduced to passive memorization of virtual or textbook images — the antithesis of “active learning increases student performance” [9].

Laboratory hours are unlikely to return to previous levels. In response to this global crisis, faculty-created five-minute histology review videos extend laboratory instruction beyond scheduled hours, providing students with 24/7 on-demand audio-visual learning that can supplement reduced laboratory time.

### Evolution from light microscopy to virtual microscopy: the technological foundation for faculty-created videos

Histology education emerged as a discipline founded on light microscopy, incorporated into medical education by 1851 [10, 11]. All faculty-created learning materials, including lectures, lecture notes, and glass slide collections, were limited but reliable.

Virtual microscopy, incorporated into histology education by the early 2000s, represented a paradigm shift [12, 13]. Faculty-created video production and blended learning became possible, opening the door to expanding resources, both reliable faculty-created content and unreliable non-faculty sources [5, 14].

AI-assisted learning tools, accelerating rapidly after 2022, represent a second paradigm shift still unfolding in histology education. Resources have become unlimited but increasingly unreliable — from questionable YouTube content [14] to AI hallucinations [15–17], creating digital noise that makes reliable content more difficult to identify.

Faculty-created five-minute histology review videos represent a return to reliable, curriculum-aligned content, easing this filtering burden for Generation Z medical students.

### Generation Z students and digital learning preferences

Contemporary medical students, primarily Generation Z (born after 1995), bring distinctive characteristics to education. They value technology-enhanced learning — strong audio-visual learning preferences, favoring lecture-style videos, audio presentations, simulations, and case studies — over assigned readings and group exercises. They also expect mentoring and prefer face-to-face interactions over independent learning [18].

However, they face three extraordinary stresses. First, the global crisis has drastically reduced laboratory hours for hands-on learning. Second, integrated curricula compress multiple subjects in short blocks, with students reporting assessment overload, excessive learning objectives, and insufficient time [19]. Third, the exponential growth in knowledge and disruptive technological innovations [20] now presents an overwhelming number of tools — Wikipedia, histology websites, spaced repetition apps (Anki), Complete Anatomy, AI assistants (ChatGPT, Gemini, Perplexity), and AI summaries (NotebookLM) — with varying reliability students cannot easily assess. Their learning preferences, combined with drastically reduced time, compressed curricula, and information overload, drive them toward review videos, including YouTube [21] and short TikTok formats. The #MedEd hashtag alone has garnered millions of TikTok views [22], underscoring their attraction to brief audio-visual formats.

Faculty-created five-minute histology review videos directly address all three realities: extending limited laboratory hours, enabling flexible on-demand study within compressed curricula, and offering reliable faculty-curated content as a trusted alternative in an era of digital noise.

### Microlearning evidence

Microlearning typically involves short, focused learning units designed to support active and flexible learning [23]. Splitting content into small pieces creates manageable learning steps; reviewing these repeatedly over time can improve knowledge retention and productivity [24]. Engagement declines with video length. Near-complete engagement was observed for videos shorter than six minutes [25], and videos under five minutes were identified as optimal for student engagement and retention in medical education [26]. In 2007, Professor John Minarcik pioneered short web-based histology videos with his Shotgun Histology collection of three-to-five-minute presentations, still available on YouTube [27]. His work demonstrated that virtual microscopy’s digital capabilities could be applied to microlearning.

Faculty-created short histology videos have been delivered across multiple platforms. YouTube-based videos augmented histology lessons in a blended approach with positive effects on student performance [28], though unvetted YouTube content varies significantly in quality and accuracy [14]. Instagram-based histology activities demonstrated significant improvements in examination performance [29]. Institutional platform videos demonstrated pre-post knowledge differences and improved self-efficacy [30]. An integrative review reported considerable impact of digital platforms, mobile apps, and virtual microscopy on histology learning [31].

Beyond histology, faculty-created short videos demonstrated positive student perceptions in gross anatomy [32], were associated with improved short-term knowledge acquisition in neuroanatomy [33], improved understanding in biochemistry [34], and were strongly associated with improved cognitive outcomes in pharmacology education [35].

Based on this evidence base, faculty-created five-minute histology review videos were anticipated to yield similar outcomes in normal histology education.

### Convergence

In the current global histology crisis, these four realities converge — histology’s visual nature, technological advances, Generation Z’s expectations, and microlearning evidence. Their precise intersection opens the door for the development and implementation of faculty-created five-minute histology review videos.

### Research gaps

Research on microlearning in health education is limited and has not established effectiveness at Kirkpatrick levels 3–4 (behavior and results) [36]. In health professions education, a systematic scoping review identified a small number of microlearning studies from a large reference pool: 94% assessed student reactions (level 1), 82% evaluated knowledge acquisition (level 2), only 29% examined behavior changes (level 3), and none measured results (level 4) [37].

No studies have systematically investigated microlearning specifically for normal medical histology. This leaves institutions worldwide without evidence-based guidance for sustainable responses to curriculum compression.

### Study purpose

This study answers the call for research into higher-level microlearning outcomes [37] by quantitatively and qualitatively examining faculty-created five-minute histology review videos designed to enhance tissue and cell identification skills among first-year medical students.

### Hypotheses

Four hypotheses were tested across Kirkpatrick evaluation levels 1–3, following the chronological sequence of the study design [38].

- H1: Students would show higher performance on tissue identification (level 2: learning)
- H2: Students would voluntarily utilize videos for examination preparation (level 3: behavior)
- H3: Students would report positive satisfaction with videos (level 1: reaction)
- H4: Students would adopt videos as a preferred learning method (level 3: behavior)

### Five theoretical foundations

Five theoretical frameworks guided the design and interpretation of this histology microlearning study. Together, they provide theoretical lenses for addressing the distinct challenges of histology learning — the intrinsic visual complexity of tissue and cell identification and the current external pressures of curriculum compression and Generation Z student needs. The collective application of all five frameworks to histology microlearning is novel.

 Cognitive load theory (CLT) states that working memory has limited capacity for processing information; instructional design must manage intrinsic load and minimize extraneous load to optimize learning [39, 40] — essential when novice learners encounter multiple unfamiliar tissues simultaneously. CLT has been applied in histology education to guide the design of multimedia learning tools [41].

 Multimedia learning theory states that “people learn more deeply from words and pictures than from words alone” [42] — foundational for histology where tissue and cell identification requires simultaneous visual and verbal processing. This theory has been applied in histology education through the design of interactive e-learning software [41].

 Variation theory states that learning occurs through systematic comparison of similarities and differences [43] — directly applicable to histology’s requirement to distinguish tissues and cells with overlapping morphological characteristics. To the best of the author’s knowledge, this is the first application of Variation Theory to histology video education in the literature, representing a novel contribution of this study.

 Spaced repetition theory states that information is better retained when reviewed at increasing intervals (distributed viewing) rather than in a single session (massed viewing) [44] — critical for histology retention in compressed curricula where natural spaced review is no longer guaranteed. Spaced education has been shown to improve long-term retention of histopathology diagnostic skills [45].

 Self-regulated learning (SRL) theory states that students independently manage their learning strategies and pace through planning, monitoring, and evaluation [46] — particularly relevant when laboratory hours are insufficient and students must take ownership of learning tissue and cell identification. To the best of the author’s knowledge, this is the first application of SRL theory to histology video education in the literature, representing a novel contribution of this study.

## Methods

### Study design and evaluation

This two-month study evaluated the effectiveness of faculty-created five-minute histology review videos among first-year medical students at Alfaisal University, Saudi Arabia. The longitudinal design followed the same student cohort across three time points within the Fall semester of 2023. The study employed a quasi-experimental pre-post within-subjects design with mixed methods, consisting of eight phases:

### Cardiovascular System (CVS) mock-OSPE (quiz 1 + video + quiz 2) (october 24–26, 2023): each laboratory group completed phases 1–6 consecutively in a single 35-minute face-to-face histology review laboratory session

- Phase 1: Pre-intervention mock-OSPE quiz (Quiz 1) — students completed 12 CVS blood vessel histology questions across six OlyVIA virtual microscopy screenshots, each accompanied by two questions, via Google Forms on own devices (Supplementary File S1). Blood vessel identification was selected based on the author's observation that it presents one of the greatest identification challenges for first-year students. Students had completed lectures and laboratory sessions on blood vessel classification prior to this assessment.
- Phase 2: Video intervention — students watched the Talking Blood Vessels video (6:02 minutes), covering blood vessel classification, structure, and function.
- Phase 3: Post-intervention mock-OSPE quiz (Quiz 2) — identical to pre-intervention quiz (Google Forms, own devices), administered immediately after the video viewing with no interval between pre- and post-intervention quizzes.
- Phase 4: Instant score feedback — students viewed their pre- and post-intervention scores instantly on their own devices.
- Phase 5: CVS Immediate survey (Survey 1) — assessing perceptions of the Talking Blood Vessels video and in-class and out-of-class learning preferences, administered immediately after Phase 3 (October 24–26, 2023) (n=193, Google Forms, own devices).
- Phase 6: Instant quiz feedback — the review laboratory session culminated in the author explaining the correct answers for all 12 mock-OSPE questions.

### Following the mock-OSPE: students gained 24/7 on-demand access to faculty-created histology review videos and completed two post-final examination surveys

- Phase 7: 24/7 on-demand access: 5 CVS videos (November 6 – November 27, 2023; 3 weeks) + 6 Renal videos (December 19 – December 26, 2023; 1 week) (Table 1). No mock-OSPE was conducted for the renal block due to time constraints within the renal curriculum.
- Phase 8: Two additional surveys via Google Forms, assessing video usage, perceptions, and in-class and out-of-class learning preferences (Survey 1 format plus video usage data).Survey 2 was administered after the CVS post-final examination (December 20, 2023 – January 14, 2024) (n=111, own devices). 

**Table 1:** List of histology review videos

Cardiovascular System Videos (*n* = 5) Access: November 6 – November 27, 2023		
Video Title	**Key Concepts Covered**	**Duration** **(min: sec)**
1. Talking Cardiac Muscle	Contractile and Non-contractile Cardiac muscle cells	5:04
2. Talking Endocardium and Epicardium	Layers of the heart, Purkinje fibers, Mesothelium	4:51
3. Talking Blood Vessels	Four types of Arteries, Structure, Function, Location, Number	6:02
4. Talking Capillaries	Types of Capillaries, Permeability	4:47
5. Talking Veins	Veins, Structure, Function, Location, Number	5:22

Renal System Videos (*n* = 6) Access: December 19 – December 26, 2023		
Video Title	**Key Concepts Covered**	**Duration** **(min: sec)**
1. Talking Kidney Cortex Histology	Nephron, Glomeruli, Tubules, Cell Types, Glomerular Capillary, Peritubular capillary	5:45
2. Talking Kidney Renal Corpuscle	Filtration Membrane, Juxtaglomerular Apparatus, Bowman’s Capsule	4:54
3. Talking Kidney Papilla-Minor Calyx Junction	Papillary Collecting Ducts, Principal Cells, Vasa Recta	5:18
4. Talking Kidney Collecting Ducts	Cortical, Medullary and Papillary Collecting Ducts	5:22
5. Talking Kidney Blood, Hormones, Filtration	Renal Blood Flow, Hormonal Control, Filtration	4:55
6. Talking Lower Urinary System	Ureters, Bladder, Urethra, Micturition	5:27

Survey 2 was administered after the CVS post-final examination (December 20, 2023 – January 14, 2024) (n=111, own devices). Survey 3 was administered after the Renal post-final examination (January 26, 2024 – February 11, 2024) (n=185, own devices).

### Kirkpatrick synthesis of phases 1–8

The evaluation followed three levels of Kirkpatrick’s framework [38] in the chronological sequence of the study design. Level 2 (Learning) was assessed through pre-post mock-OSPE quizzes on blood vessel identification. Level 3 (Behavior) was measured through video usage patterns and student preference and adoption. Level 1 (Reaction) was evaluated through student perceptions collected via surveys. Level 4 (Results) was not assessed, as it was beyond the scope of this study.

### Setting and participants

The study was conducted at Alfaisal University College of Medicine, Riyadh, Saudi Arabia, during the 2023–2024 academic year. Histology is taught as part of an integrated systems-based curriculum in the first year, Fall semester. Face-to-face instruction consists of approximately two lecture hours per week and one 35-minute histology rotation within a multi-station anatomical sciences laboratory. The curriculum provides multiple teaching modalities: histology lectures, face-to-face classical microscopy, team-based learning (TBL), peer teaching in evening labs, review open laboratory sessions, discussion with histology teachers, OlyVIA virtual microscopy, and faculty-created short videos.

The CVS module cohort comprised 474 first-year medical students (190 male, 284 female) and the Renal module cohort comprised 481 students (192 male, 289 female). The Renal module cohort was slightly larger than the CVS cohort. Cohort numbers reflect students who sat each module examination. At Alfaisal University, students may skip a module examination or retake a previously failed examination, resulting in cohort size differences between modules.

A total of 193 students participated in the mock-OSPE during a regularly scheduled histology laboratory session on CVS histology review (40.7% of CVS cohort). Survey 2 was completed voluntarily after the CVS post-final examination by 111 students (23.4%); Survey 3 was completed voluntarily after the Renal post-final examination by 185 students (38.5%).

### Ethical considerations

This study received approval from the Institutional Review Board at Alfaisal University (reference number IRB-18076). Participation in the mock-OSPE quizzes and three surveys was voluntary. All data were collected anonymously and kept confidential. Students provided informed consent electronically through Google Forms distributed by email prior to completing the quizzes and surveys.

### Interventions

As a histology faculty member at Alfaisal University, the author created 35 five-minute histology review videos for first-year medical students covering all organ systems in the integrated preclinical medical curriculum.

### Slide digitization

Alfaisal University’s collection of glass microscope slides was sourced from multiple suppliers: Carolina Company (USA), Sultan Qaboos University (Oman), and Johannes Lieder (Germany). High-quality, well-stained slides were selected and digitized using the Olympus VS120 Virtual Slide Scanner. Digitized slides were labeled and uploaded to Alfaisal University’s digital histology library cloud storage.

### Student access

Students downloaded the digitized slides from Alfaisal University’s digital histology library cloud storage and installed OlyVIA software (version 2.9.1), a free virtual microscopy viewer from Olympus. OlyVIA enabled students to view and interact with digitized slides on their laptops, replicating traditional light microscope functions including slide navigation and zooming. Additional digital capabilities beyond traditional microscopy included simultaneous comparison of two to four slides, pre-existing faculty labels, and the ability to create personal labels.

### Video storyboard development, recording and distribution

 The video production process is summarized in Table 2.

**Table 2:** Video Storyboard Development, Recording and Distribution

Step	Description
Step 1	The best quality digitized slides of each block were selected.
Step 2	Within each slide, the best quality tissue regions were identified for video recording purposes.
Step 3	Selected regions were captured as screenshots.
Step 4	Screenshots were imported into PowerPoint presentations.
Step 5	Screenshots were arranged systematically by magnification level: low magnification (40×): whole organ overview; medium magnification (100×): tissue layers; high magnification (400×): cellular details.
Step 6	PowerPoint slides were labeled with key histological terminology.
Step 7	Narration scripts were written for each video.
Step 8	Narration scripts were rehearsed repeatedly and edited as needed to maintain the five-minute format prior to recording. Videos exceeding a 6.5-minute threshold were re-recorded.
Step 9	Videos were recorded using Screencast-O-Matic from 2015, transitioning to Zoom screen recording in 2020. All videos were created in audio-visual format, incorporating three visual elements — microscopic images, labeled terminology, and cursor movements — synchronized with narration.
Step 10	Completed videos were shared with students via Google Drive links distributed by email.

Steps 1–4 were performed using OlyVIA

Steps 5–6 were completed in PowerPoint

### Video titles

Videos were labeled with the prefix “Talking” to distinguish narrated videos from standard PowerPoint lecture slides (e.g., “Talking Blood Vessels,” “Talking Kidney”) (Table 1).

### Data collection

Data were collected using five instruments administered via Google Forms. The first instrument was the pre-intervention mock-OSPE quiz (Quiz 1). The second instrument was the post-intervention quiz (Quiz 2). The third instrument was the CVS Immediate survey (Survey 1). The fourth instrument was the CVS post-final examination survey (Survey 2). The fifth instrument was the Renal post-final examination survey (Survey 3). Administration dates and details are described in Methods: 1. Study Design and Evaluation section above.

All survey instruments were developed by the author and are provided in Supplementary File S2. Open-ended qualitative comments were collected through all three surveys. Supplementary File S2 — Data Collection Instruments.

### Statistical analysis

Data were analyzed using three tools: jamovi (version 2.3.21), Microsoft Excel, and an online statistical calculator (socscistatistics.com). Descriptive statistics including means, medians, standard deviations, and percentages were calculated for all variables using Microsoft Excel. Pre-test and post-test mock-OSPE scores across 12 questions were analyzed in jamovi using a two-tailed paired samples t-test, with normality confirmed using the Shapiro-Wilk test. Cohen’s d and 95% confidence intervals for mean differences were computed in jamovi. Survey responses were compared using chi-square tests with Cramér’s V as the effect size measure using socscistatistics.com. Statistical significance was set at α = 0.05.

## Results

### Hypothesis 1: mock-OSPE performance

First-year medical students completed pre- and post-intervention quizzes (Quiz 1, Quiz 2) on 12 blood vessel histology questions after a single video intervention. Performance was significantly higher in the post-intervention assessment, with a large effect size (Table 3, Supplementary Figure S3). Higher scores were observed across eleven of twelve question pairs, with the highest occurring in large elastic artery, resistance artery, and small muscular artery identification. One question score showed a minimal decrease: endothelium identification.

**Table 3:** CVS Mock-OSPE Performance

Multiple Choice Questions One answer is accepted	Correct Answer	Before Video % (*N* of Total)	After Video % (*N* of Total)	Change (% points)
1. What is the structural name of this blood vessel?	Large elastic artery	31.1% (56 of 180)	63.8% (118 of 185)	+ 32.7
2. Give the name of the cell at the arrow?	Endothelium	60.0% (108 of 180)	58.8% (110 of 187)	-1.2
3. What is the structural name of this blood vessel at image B?	Small muscular vein	18.4% (34 of 185)	36.0% (68 of 189)	+ 17.6
4. What is the tissue at the arrow?	Connective tissue	34.8% (63 of 181)	42.9% (81 of 189)	+ 8.1
5. What is the functional name of this artery?	Resistance artery	28.7% (54 of 188)	56.7% (106 of 187)	+ 28.0
6. How many layers of smooth muscle can you see?	2	60.2% (112 of 186)	71.3% (134 of 188)	+ 11.1
7. What is the name of the layer at the arrow?	Tunica media	60.2% (112 of 186)	78.2% (147 of 188)	+ 18.0
8. What type of cells can you see in this layer?	Only smooth muscle	33.5% (62 of 185)	53.2% (100 of 188)	+ 19.7
9. What is the structural name of this vessel?	Small muscular artery	25.8% (48 of 186)	49.7% (95 of 191)	+ 23.9
10. What is the key identification point?	1 fenestrated elastic lamina	31.9% (59 of 185)	50.3% (95 of 189)	+ 18.4
11. In which layer can you see this vessel at the arrow?	Tunica externa	58.9% (109 of 185)	60.3% (114 of 189)	+ 1.4
12. What is the function of this vessel?	Vasa vasorum	26.5% (49 of 185)	28.7% (54 of 188)	+ 2.2
**Overall**		M = 39.2%, SD = 15.8	M = 54.2%, SD = 14.0	Mean diff = 15.0% points

Statistics: t(11) = 4.80, *p* = 0.001, Cohen’s d = 1.39, 95% CI [8.12, 21.86]. Shapiro-Wilk normality confirmed: W = 0.955, *p* = 0.705.

All items were single-answer multiple-choice questions (one correct answer from five options). N per item reflects students who responded to that item; students who did not respond to an item were excluded from that item’s analysis

Supplementary Figure S3 — Bar Graph of Mock-OSPE Performance Before and After Video Intervention.

Supplementary Figure S3 caption: Percentage of correct answers per question before (blue) and after (orange) the Talking Blood Vessels video intervention. Questions 1–12 are individual histology identification items. Response rates varied per item; n per question is reported in Table 3.

### Hypothesis 2: voluntary utilization

Students showed high voluntary usage across both organ systems. The majority of students in both cohorts watched the videos at least twice. The median number of views was identical across cohorts. Video viewing frequency did not differ significantly between cohorts (Table 4). When normalized for access period duration, students watched at a higher weekly rate during the Renal module than the CVS module.

**Table 4:** Video Viewing Frequency

Number of Times Watched	CVS Videos (*n* = 111)	Renal Videos (*n* = 185)
0	3.6% (4)	8.1% (15)
1	10.8% (12)	21.1% (39)
2	42.3% (47)	30.3% (56)
3	20.7% (23)	20.0% (37)
4	7.2% (8)	6.5% (12)
5+	15.3% (17)	14.1% (26 )
Mean (range)	2.63	2.38
Median	2	2
Normalized (views/week)	0.88	2.38

Statistics: χ² (5) = 9.49, *p* = 0.091, Cramér’s V = 0.179

Video viewing timing did not differ significantly between cohorts (Table 5). In both cohorts, students predominantly watched the videos either the night before the examination or throughout the entire access period. Students in the Renal module cohort showed a slightly higher tendency to watch throughout the access period, while students in the CVS module cohort showed a slightly higher tendency to watch the night before the examination.

**Table 5:** Video Viewing Timing

View Timing (One answer is accepted)	CVS Videos (*n* = 107)	Renal Videos (*n* = 175)
Immediately	7.5% (8)	13.1% (23)
Night before exam	45.8% (49)	40.6% (71)
Throughout the entire given time	39.3% (42)	44.6% (78)
Never	0.9% (1)	0.6% (1)
Other	6.5% (7)	1.1% (2)

Statistics: χ² (4) = 9.00, *p* = 0.061, Cramér’s V = 0.179

### Hypothesis 3: student perceptions

Response rates were substantially higher for positive perception questions (Table 6) than for negative perception questions (Table 7) across all three time points. Among students who responded to the negative perception questions, a few provided positive free-text comments in the negative question field. Six of ten positive perception features changed significantly across time points (Table 6). The two aspects showing the largest and most consistent increases across all three time points were the concise five-minute length and the simplified presentation of complex concepts. Side-by-side structural comparison showed a sustained increase from immediate to post-examination time points. Other aspects, including the ability to pause and rewind and the combination of sensory modalities, increased at the CVS post-final examination time point but did not sustain this level at the Renal post-final examination time point (Table 6).

**Table 6:** Positive Perceptions of Videos

Positive Aspects (Select up to 3)	CVS Immediate (*n* = 184)	CVS Post-Final Examination (*n* = 103)	Renal Post-Final Examination (*n* = 172)	Statistics Across Timepoints
Appreciate learning through listening rather than reading	44.6% (82)	48.5% (50)	55.2% (95)	χ²(2) = 4.09, *p* = 0.129, V = 0.094
Video’s concise length (5 min) minimized distractions	54.9% (101)	71.8% (74)	73.3% (126)	χ²(2) = 15.59, *p* < 0.001, V = 0.184
Presented complex concepts in simplified, easy-to-understand manner	46.7% (86)	68.0% (70)	64.0% (110)	χ²(2) = 16.27, *p* < 0.001, V = 0.188
Effectively compared structures side by side for better understanding	26.6% (49)	41.7% (43)	41.9% (72)	χ²(2) = 11.07, *p* = 0.004, V = 0.155
Hearing key medical terminologies was beneficial	14.7% (27)	23.3% (24)	23.3% (40)	χ²(2) = 5.13, *p* = 0.077, V = 0.106
Combination of seeing, reading, listening, and visual aids enhanced learning	37.5% (69)	46.6% (48)	41.9% (72)	χ²(2) = 2.31, *p* = 0.315, V = 0.071
Allowed skipping histology book reading, saving time	21.7% (40)	37.9% (39)	36.0% (62)	χ²(2) = 11.74, *p* = 0.003, V = 0.160
Provided entertaining and educational experience simultaneously	19.0% (35)	25.2% (26)	24.4% (42)	χ²(2) = 2.09, *p* = 0.352, V = 0.067
Can pause, rewind, replay, and access conveniently anywhere	37.5% (69)	52.4% (54)	41.9% (72)	χ²(2) = 6.07, *p* = 0.048, V = 0.115
Utilized screenshots of microscopic slides from histology laboratory	17.9% (33)	28.2% (29)	29.1% (50)	χ²(2) = 6.99, *p* = 0.030, V = 0.123
Response rate	95.3% (184 of 193)	92.8% (103 of 111)	93.0% (172 of 185)	

Positive vs. negative response rates across timepoints: χ² (2) = 27.36, *p* < 0.001, Cramér’s V = 0.237 (also reported in Table 7)

Due to the low response rate for negative perception questions, findings are descriptive only. Worry about OSPE sufficiency was the most prominent negative finding at both time points, with a higher proportion reporting this in the Renal post-final examination survey. In that same survey, some students also reported not typically studying with videos. A few students who responded to the negative perception questions provided positive free-text comments (Table 7).

**Table 7:** Negative Perceptions of Videos

Negative Perceptions (Select up to 3)	CVS Immediate (*n* = 106)	CVS Post-Final Examination (*n* = 26)	Renal Post-Final Examination (*n* = 35)
Prefer learning through reading	6.6% (7)	3.8% (1)	11.4% (4)
Do not typically study with videos	0.9% (1)	0.0% (0)	25.7% (9)
Prefer interactive learning experiences rather than passive video watching	14.2% (15)	7.7% (2)	22.9% (8)
Video lacked engaging or captivating elements	1.9% (2)	7.7% (2)	11.4% (4)
Video was too fast-paced	7.5% (8)	7.7% (2)	2.9% (1)
Prefer longer and more comprehensive explanations	8.5% (9)	0.0% (0)	5.7% (2)
Concerned that information provided might be incomplete	3.8% (4)	7.7% (2)	11.4% (4)
Worried it would not be sufficient for an OSPE	5.7% (6)	15.4% (4)	34.3% (12)
Response rate	54.9% (106 of 193)	23.4% (26 of 111)	18.9% (35 of 185)

Positive vs. negative response rates across timepoints: χ²(2) = 27.36, *p* < 0.001, Cramér’s V = 0.237 (also reported in Table 6)

Statistics Across Timepoints: not calculated due to insufficient responses. Descriptive findings are reported for exploratory purposes only

### Hypothesis 4: learning preferences and video adoption

In-class learning preferences showed distinct patterns across three teaching modalities: face-to-face microscopy, mock-OSPE, and histology lecture attendance. Face-to-face classical microscopy was selected by the highest number of students at both post- examination time points. Mock-OSPE was the second most preferred. Histology lecture attendance was the least preferred in-class activity, though attendance increased significantly between the two post-final examination time points (Table 8).

**Table 8:** In-Class Learning Preferences

Teaching Activities (Select up to 3)	CVS Immediate (*n* = 190)	CVS Post-Final Examination (*n* = 107)	Renal Post-Final Examination (*n* = 183)	Statistics Across Post-Final Examination Timepoints
Histology lecture	15.8% (30)	29.0% (31)	44.8% (82)	χ²(1) = 6.47, *p* = 0.011, V = 0.149
Histology, face-to-face classical microscopy	27.4% (52)	79.4% (85)	70.5% (129)	χ²(1) = 2.35, *p* = 0.125, V = 0.090
TBL	17.9% (34)	37.4% (40)	44.8% (82)	χ²(1) = 1.24, *p* = 0.266, V = 0.065
Peer teaching in evening labs	10.5% (20)	23.4% (25)	24.0% (44)	χ²(1) = 0.00, *p* = 1.000, V = 0.000
Mock-OSPE	20.5% (39)	54.2% (58)	53.6% (98)	χ²(1) = 0.00, *p* = 1.000, V = 0.000
Discussion with histology teachers	7.9% (15)	47.7% (51)	38.8% (71)	χ²(1) = 1.83, *p* = 0.176, V = 0.079

CVS Immediate survey questions were configured as single-select (one response permitted). CVS Post-Final Examination and Renal Post-Final Examinationsurveys were configured as multi-select (up to 3 responses permitted). Statistics column compares CVS Post-Final Examination vs. Renal Post-Final Examination only

Out-of-class study method preferences showed distinct patterns across three categories: reading PowerPoint lectures, question banks, and faculty-created short videos. Reading PowerPoint lectures was consistently the most selected out-of-class study method at both post-final examination time points. Question banks were highly selected as an out-of-class study method. Faculty-created short videos reached nearly the same level as reading PowerPoint lectures through a 17-fold increase in adoption across time points (Table 9).

**Table 9:** Out-of-Class Learning Preferences

Study Methods (Select up to 3)	CVS Immediate (*n* = 190)	CVS Post-Final Examination (*n* = 110)	Renal Post-Final Examination (*n* = 185)	Statistics Across Post-Final Examination Timepoints
Active Study Methods				
Reading Histology Textbook	0.5% (1)	3.6% (4)	2.7% (5)	χ²(1) = 0.01, *p* = 0.920, V = 0.006
Reading PowerPoint lecture	71.6% (136)	81.8% (90)	83.8% (155)	χ²(1) = 0.08, *p* = 0.784, V = 0.016
OlyVIA virtual microscopy	1.1% (2)	8.2% (9)	9.2% (17)	χ²(1) = 0.01, *p* = 0.934, V = 0.005
Faculty-created short videos	4.7% (9)	81.8% (90)	81.6% (151)	χ²(1) = 0.00, *p* = 1.000, V = 0.000
YouTube videos	8.4% (16)	13.6% (15)	17.3% (32)	χ²(1) = 0.44, *p* = 0.505, V = 0.039
Question banks	11.1% (21)	66.4% (73)	64.9% (120)	χ²(1) = 0.02, *p* = 0.892, V = 0.008
Internet resources	2.6% (5)	9.1% (10)	11.9% (22)	χ²(1) = 0.31, *p* = 0.579, V = 0.032

CVS Immediate survey questions were configured as single-select (one response permitted). CVS Post-Final Examination and Renal Post-Final Examination surveys were configured as multi-select (up to 3 responses permitted). Statistics column compares CVS Post-Final Examination vs. Renal Post-Final Examination only

## Discussion

### Main findings

This study addresses the limited evidence base for microlearning in medical histology education. Recent systematic reviews screened 3,188 studies from 2020 to 2024 [47]. Forty studies met criteria across various disciplines; only five addressed medical education. Objective learning outcomes remain underexplored. To address this gap, rigorous evidence was gathered across three Kirkpatrick levels using both objective and self-reported measures.

The four hypotheses were supported with the following findings. One brief faculty-created video correlated with a measurable score increase in the mock-OSPE blood vessel identification assessment. The majority of students watched the videos multiple times across both organ systems, consistent with voluntary and repeated engagement. Positive perceptions remained consistently high across all three time points. The 17-fold increase in video adoption represented an exceptional behavioral shift in microlearning research on histology education.

Two hypotheses addressed Kirkpatrick Level 3 behavior — voluntary video utilization and preferred method adoption — directly responding to the identified gap in behavioral outcome evidence in microlearning research [37]. The convergence of objective and self-reported findings is consistent with the conclusion that faculty-created five-minute histology review videos function as an effective microlearning tool for histology education.

### Hypothesis 1: mock-OSPE Performance (Kirkpatrick level 2 - learning)

During the mock-OSPE (Quiz 1 + video + Quiz 2), students’ performance was significantly higher in the post-intervention assessment after a single video viewing. Comparable gains in osteopathic medical education required supplementary video access across two years of curriculum in three consecutive cohorts [48]. At Alfaisal University, a single viewing of a faculty-created five-minute histology review video correlated with a measurable score increase with minimal exposure.

The effect size was large and educationally meaningful, not just statistically significant. This exceeds prior video-based learning benchmarks in medicine for both knowledge acquisition and skills development [49]. The lower bound of the confidence interval alone is consistent with a substantial increase in post-intervention scores. The large effect size (Cohen’s d = 1.39) further supports the conclusion that the score difference exceeds what would be expected from question and image familiarity alone.

Higher scores were observed across eleven of twelve question pairs, with the highest occurring in large elastic artery, resistance artery, and small muscular artery identification — highest quality slides with clear visualization of identification criteria, which the video addressed directly. In contrast, the least pronounced score increases occurred in questions requiring identification of specific layers, tissues, and cell types within vessels. This suggests that a single video viewing was more effective for vessel-level recognition than for histological details of the blood vessel wall, which may require repeated viewing or additional instructional support.

One question score showed a minimal decrease: endothelium identification. While the difference was negligible, the pre-test performance was also unexpectedly low for a structure that lines all blood vessels and serves as a key identification point across every vessel type. Endothelium should be among the most recognizable features precisely because of its ubiquity. The reason for this pattern remains unclear and warrants further investigation — whether it reflects a gap in prior teaching, difficulty distinguishing endothelium from surrounding cells at higher magnification, or limitations of a single video viewing for consolidating this level of discrimination.

The mock-OSPE exposed knowledge gaps for both students and the author. Students could self-correct immediately and redirect their study focus, while the author could analyze the underlying causes of these gaps and adjust instructional priorities.

To the best of the author’s knowledge, no similar mock-OSPE format combining video intervention with pre-post assessment, quantitative instant score feedback, and qualitative instant quiz feedback has been reported in histology microlearning literature.

Hypothesis 1 was supported — a single viewing of a faculty-created five-minute histology review video was associated with a statistically significant increase in tissue identification performance.

### Hypothesis 2: voluntary utilization (Kirkpatrick level 3 - behavior)

Students showed similar voluntary engagement with faculty-created histology review videos across both CVS and Renal curricular blocks. Video usage was high among respondents across both organ systems. Most students watched the videos multiple times. The difference in viewing frequency between organ systems reflects access duration. When normalized for access time, Renal module students watched the videos more frequently per week than CVS module students. 

Repeated viewing was a design goal, and many students used this feature. This aligns with spaced repetition principles — online spaced education has been shown to generate transfer and substantially improve long-term retention of diagnostic skills compared to single exposure, including in histopathology [44, 45].

Viewing timing patterns revealed a descriptive difference between organ systems, though this did not reach statistical significance. Students with three-week access to CVS videos showed a higher tendency toward massing into last-minute viewing — more watched the night before the examination than throughout the access period. Students with one-week access to Renal videos showed a higher tendency toward distributed viewing — more watched throughout the access period than the night before. Shorter access windows may discourage procrastination.

Prior review papers reported the absence of behavioral outcome data in microlearning research [36, 37]. Findings from Alfaisal University on voluntary utilization help address this gap, as reflected in two complementary measures: viewing frequency and viewing timing patterns.Three behavioral findings — voluntary usage, multiple viewing, and viewing timing patterns — together reflect behavior change at Kirkpatrick Level 3, beyond Level 1 satisfaction measures. These patterns replicated across both organ systems, suggesting findings are generalizable rather than topic-specific.

Hypothesis 2 was supported — students voluntarily utilized faculty-created five-minute histology review videos for examination preparation across both organ systems.

### Hypothesis 3: student perceptions (Kirkpatrick level 1 - reaction)

Positive perceptions outweighed negative ones throughout the study. Student satisfaction remained consistently high across all three time points. Positive perceptions across ten video features were higher at post-examination time points than at the immediate time point, while negative perceptions across eight features remained low.

The two most consistently appreciated features were concise length with minimal distractions and simplified presentation of complex concepts — mapping directly to Microlearning theory and Cognitive Load Theory respectively [39]. The third most appreciated feature was side-by-side structural comparison, which showed a sustained increase across all post-examination time points. This feature maps to Variation Theory [43] — students can visually analyze similar-looking tissues simultaneously, a benefit not consistently available in generic YouTube histology videos. The relatively low initial ranking of this feature may reflect limited student awareness of comparison as a deliberate learning strategy — a benefit intentionally built into the videos that students only gradually recognized with repeated use.

Students also valued control over viewing — pause, rewind, replay, and convenient access — and the combination of seeing, reading, listening, and visual aids, consistent with Multimedia Learning Theory [42]. Both features increased at the CVS post-final examination time point but did not sustain this level at the Renal post-final examination time point, suggesting these benefits were most appreciated when students had extended access. One student reported watching at 2× speed in a free-text response, demonstrating self-paced engagement beyond standard viewing.Most students had nothing negative to say — they simply did not engage with the negative perception question, suggesting an active avoidance of negativity. Those who did respond negatively were a small and declining minority, especially apparent in view of the high number of students who engaged with positive perceptions.

Worry about OSPE sufficiency was the most prominent negative finding, especially in the Renal survey, where students seemingly lost confidence with only one week of access versus three weeks for CVS beforehand. This reflects access limitation rather than video inadequacy. In the Renal post-final examination survey, some students also reported not typically studying with videos — a prior study habit rather than a negative perception of the videos.

In free-text responses, students described the videos as making content less overwhelming and enhancing visualization, and expressed strong enthusiasm — including comments on their vital role in OSPE preparation and requests for continuation.

The progressive disengagement from negative perception questions across time points reflects a behavioral choice — students actively chose to engage with positive aspects while declining to report negative ones. A few students went further, providing positive free-text comments in the negative question field, suggesting that anticipated concerns did not materialize after encountering the videos — the negative questionnaire ended on a positive note, a finding that contributed to the behavioral evidence base in microlearning research [37].

Hypothesis 3 was supported — students reported consistently positive perceptions of faculty-created five-minute histology review videos across all three time points. 

### Hypothesis 4: Learning Preferences and Video Adoption (Kirkpatrick level 3 - behavior)

In-class learning preferences remained consistent with active learning values. Face-to-face classical microscopy remained the top in-class choice at both post-examination time points. Mock-OSPE was the second most preferred in-class activity. Both reflect students' appreciation for direct, hands-on, and active learning experiences in the histology laboratory. The substantial increase in mock-OSPE preference from immediate to post-examination time points further suggests that students valued this format once they experienced it firsthand. Histology lecture attendance was third among in-class activities, possibly reflecting the passive nature of lectures. Attendance increased significantly between the two post-examination time points, with higher rates at the Renal time point. This may reflect the compressed Renal curriculum block and the complexity of renal histology content.

Out-of-class study methods revealed the most dramatic behavioral shifts in the entire study — a crescendo across three categories. While few students attended histology lectures in class, reading PowerPoint lectures was consistently the most selected out-of-class study method — the same content students avoided in class, they embraced independently at home. Question bank usage increased markedly, possibly reflecting mock-OSPE experience — students recognized the value of question-based practice and independently adopted it as a study method.The adoption of faculty-created videos represented the largest preference change in the entire study, increasing 17-fold and reaching nearly the same level as PowerPoint lectures at both post-final examination time points. This adoption rate was identical across two consecutive modules, suggesting that direct experience with faculty-created content corresponded with a stable and reproducible change in student learning behavior. This adoption trajectory follows Rogers' Diffusion of Innovation theory [50] — within a single semester, students moved from awareness to trial to full adoption through direct experience.

These findings reveal a striking Generation Z paradox — embracing the oldest learning tool (classical microscopy) and the newest (faculty-created digital videos) simultaneously. PowerPoint lectures remained the most selected out-of-class method throughout — videos complemented rather than replaced them. Students did not abandon traditional study methods — they integrated faculty-created videos as an additional study tool. This is the expected, reported Generation Z learner profile: blended learning combining human contact inside class with digital efficiency outside class [18]. At Alfaisal University, Generation Z students unknowingly chose blended learning — a strategy that systematic reviews associate with consistently better knowledge outcomes compared to traditional instruction alone in health education [51]. Medical students in blended learning environments have been associated with self-regulated learning, improved study habits, and greater autonomy in resource selection [52]. Out-of-class and in-class preferences converged around materials created and delivered by the same histology instructor — PowerPoint lectures, laboratory sessions, open histology laboratory, TBL, faculty-created short videos, and the mock-OSPE assessment. This suggests that pedagogical coherence and trusted authorship across all modalities may drive student adoption.

In-class preferences told a preference story — active learning formats valued most. Out-of-class preferences told a behavioral change story — digital adoption climbing highest. Together they reflected the Generation Z blended learning profile.

Hypothesis 4 was supported — faculty-created five-minute histology review videos became students' preferred out-of-class learning method, representing a 17-fold behavioral shift in learning preferences.

### Kirkpatrick Evaluation Summary

Faculty-created five-minute histology review videos were effective (Level 2 — significant performance advances), replicated (Level 3 — 17-fold adoption increase in two modules), and aligned with Generation Z expectations (Level 1 — positive perceptions of face-to-face microscopy inside class and digital videos outside class) (Table 10).

**Table 10:** Summary of Kirkpatrick Evaluation Findings for Faculty-Created Five-Minute Histology Review Videos

Kirkpatrick Level	Category	Hypothesis	Key Findings
Level 1 — Reaction	Perceptions of five-minute videos	H3	• Positive perceptions consistently high across all three timepoints • Six of ten positive perception features exhibited significantly higher endorsement: largest for concise length and simplified concepts • Negative perceptions low across time points • Positive perceptions reflected Generation Z blended learning expectations: digital videos outside class and face-to-face microscopy inside class
Level 2 — Learning	Performance of Mock-OSPE after single video viewing	H1	• Scores were significantly higher in the post-intervention assessment • Large effect size (d = 1.39) • Higher scores observed in eleven of twelve questions • Largest score increases: large elastic artery, resistance artery, small muscular artery
Level 3 — Behavior	Usage of five-minute videos	H2	• Students exhibited high voluntary usage across both organ systems • Students watched videos multiple times • Students showed timing patterns reflecting access duration • Students showed low engagement with negative perception features
Level 3 — Behavior	Adoption of five-minute videos	H4	• Faculty-created videos showed a 17-fold increase in adoption • Adoption replicated across two consecutive modules • Students reached the same adoption level as PowerPoint lectures • Adoption trajectory followed Rogers’ Diffusion of Innovation within a single semester
Level 4 — Results	Institutional outcomes	—	• Not assessed — beyond scope of this study

### Five theoretical frameworks

The video design integrated all five theoretical frameworks: brief focused content minimizing processing load (Cognitive Load Theory), dual-channel processing (Multimedia Learning), side-by-side tissue comparisons (Variation Theory), repeated viewing options (Spaced Repetition), and voluntary student control (Self-Regulated Learning). Students endorsed these theory-informed design principles without recognizing them as theoretical concepts — indicating that effective instructional design works invisibly. The frameworks address five distinct dimensions of the learning challenge: how to manage cognitive load, how to engage dual sensory channels, how to teach visual discrimination, how to ensure retention through repetition, and how to enable student ownership of learning. Collectively, these five frameworks offer a practical response to curriculum compression in histology education, contributing to efficient microlearning within limited instructional time. 

#### Cognitive Load Theory

**Video Design: Reduced Cognitive Demands**

Each video was built around one learning objective, managing intrinsic load by content segmentation. Each video was designed with a five-minute duration, reducing extraneous load by minimizing distractions. Each video was recorded to highlight specific identification points — directing students' focus to critical features to free working memory for consolidation and to facilitate germane load processing [39–41].

**Student Response to Reduced Cognitive Demands**

Students' top two choices were simplified complexity (intrinsic load) and minimized distractions (extraneous load). Both were direct indicators that faculty-created five-minute histology review videos successfully diminished cognitive load.

***The Cognitive Load Theory-guided design worked.***

#### Multimedia Learning Theory

**Video Design: Dual-channel Learning**

Audio-visual design was built into every video for dual-channel brain processing [41, 42]. Each video combined synchronized narration with three visual elements — OlyVIA virtual microscopy screenshots, labeled terminology, and cursor movements.

**Student Response to Dual-channel Learning**

Dual-channel engagement was reflected in these two student choices: audio-visual preference and the combination of seeing, reading, listening, and visual aids. This result reflected that faculty-created five-minute histology review videos aligned with Generation Z’s dual-channel preferences [18].

***The Multimedia Learning Theory-guided design worked.***

#### Variation Theory

**Video Design: Comparison**

**Fig. 1:**
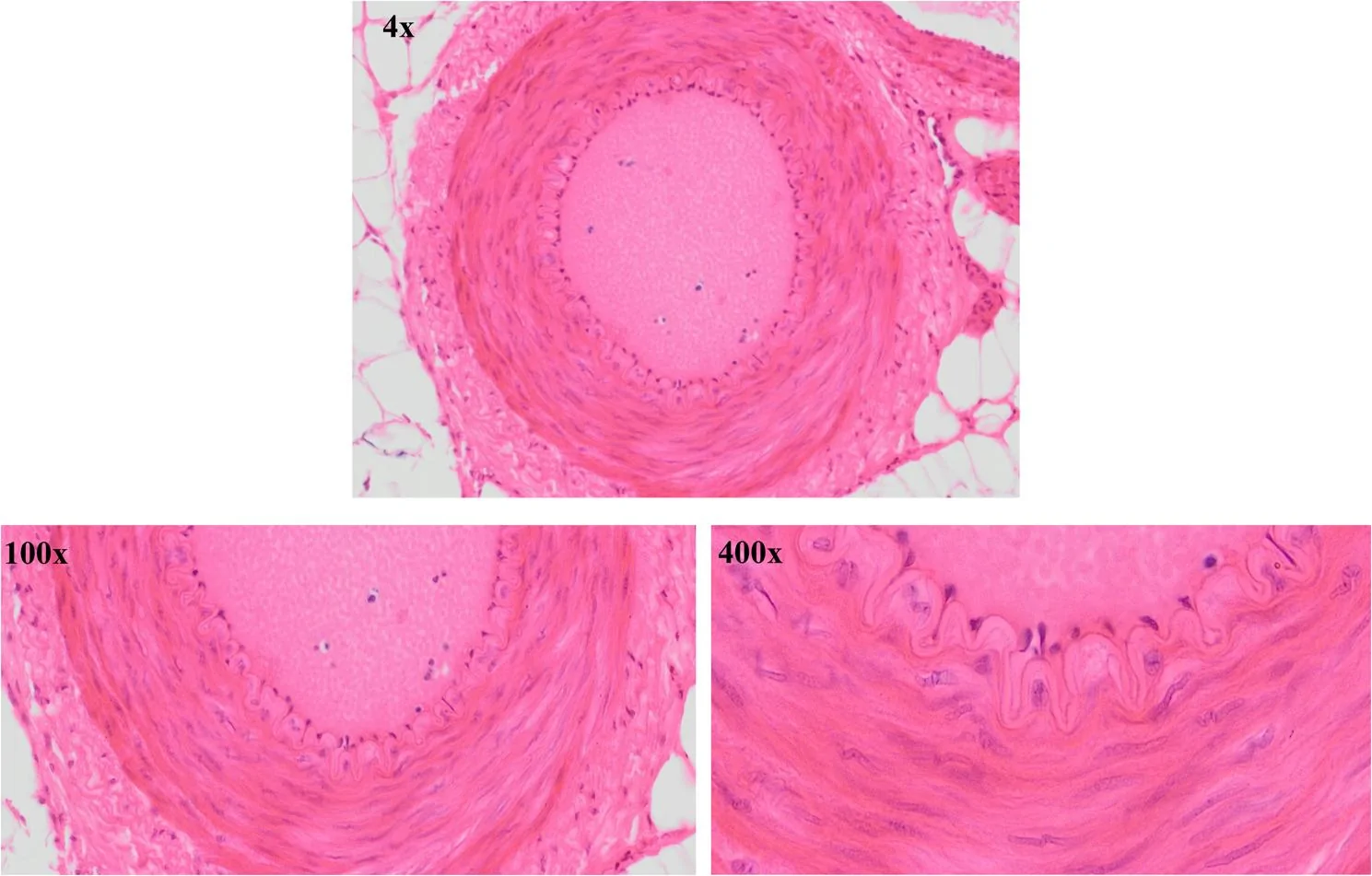
Comparison and Identification of a Blood Vessel Identification: OlyVIA virtual microscopy screenshots exhibiting a small muscular artery in three magnification levels

**Fig. 2:**
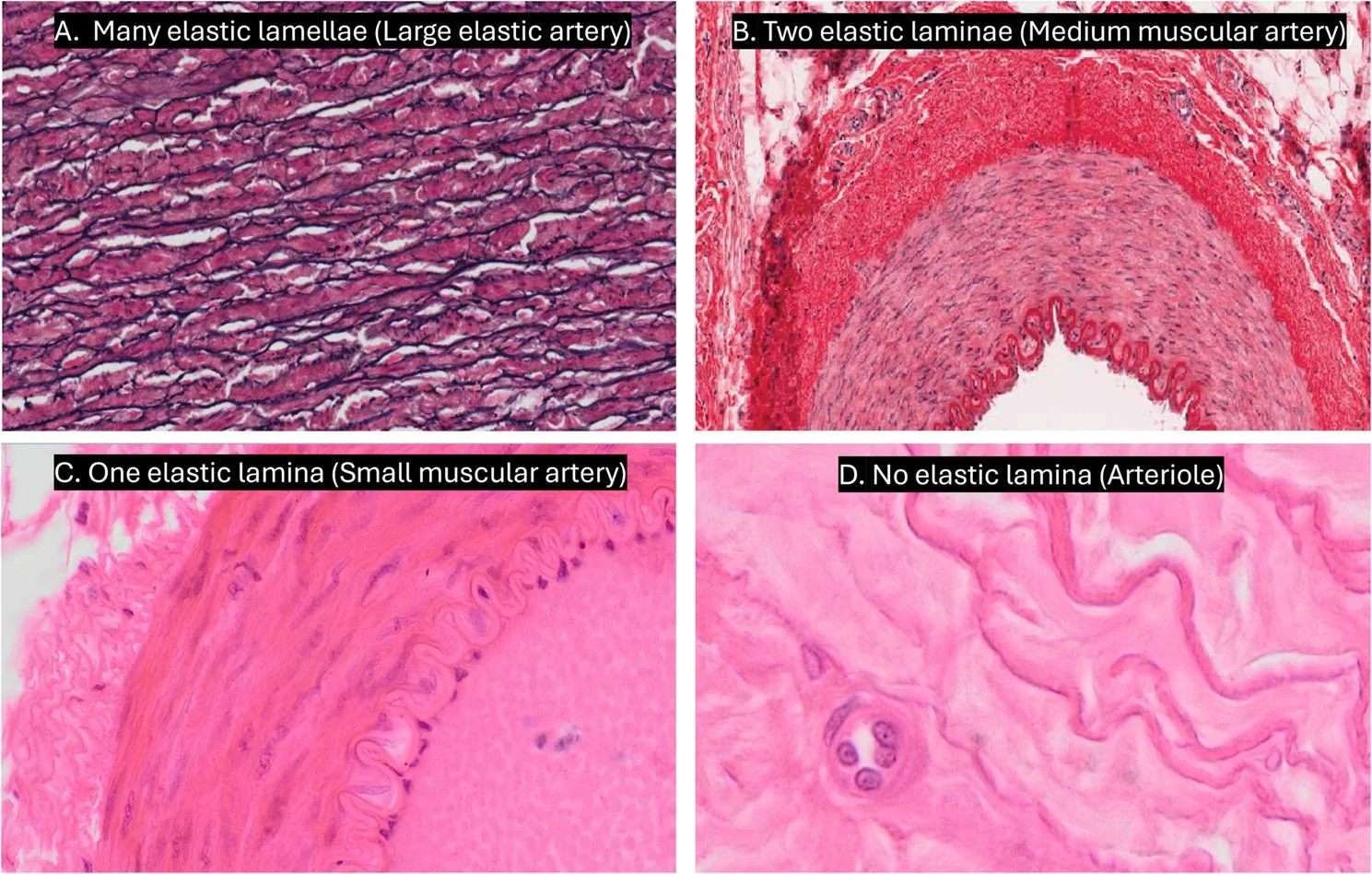
Comparison and Classification of Arteries Classification: OlyVIA virtual microscopy screenshots showing side-by-side comparison of four artery types classified by their elastic laminae.** A**. Many elastic lamellae — large elastic artery. **B**. Two elastic laminae — medium muscular artery. **C**. One elastic lamina — small muscular artery. **D**. No elastic lamina — arteriole

Videos systematically applied side-by-side comparisons of seemingly identical tissues and cells to support three levels of visual discrimination: identification across different magnifications (Fig. 1), classification across structural types (Fig. 2), and function through contrasted anatomical features and locations [43].

**Student Response to Comparison**

Students initially undervalued side-by-side comparison. Their endorsement increased and was sustained at both post-examination time points. Repeated experience with faculty-created five-minute histology review videos opened their eyes to the value of comparison.

***The Variation Theory-guided design worked — but recognition was slow.***

#### Spaced Repetition Theory

**Video Design: Brevity**

Videos were deliberately limited to five to six minutes, with 24/7 access made available via Google Drive to enable spaced repetition — if practiced, critical for histology retention [44, 45, 53]. Repeated viewing alone does not guarantee spaced repetition — timing determines whether students engaged in distributed or massed viewing. Access time must be optimal: long enough to enable spacing, short enough to discourage procrastination.

**Student Response to Brevity**

Most students watched the videos multiple times, with the majority viewing them at least twice, consistent with repeated engagement. This reflected that faculty-created five-minute histology review videos provided the conditions for spaced repetition, though spacing intervals were not directly measured.

***The Spaced Repetition Theory-guided design worked — but effectiveness depended on access duration.***

#### Self-Regulated Learning Theory

**Video Design: On-demand Access**

Videos were available 24/7 via Google Drive, without scheduling constraints. This gave students full autonomy to decide which videos to watch, when to watch them, and how often [46, 52, 54].

**Student Response to On-demand Access**

Students engaged with all three SRL components: choosing when to watch (planning), repeated viewing (monitoring), and shifting to faculty-created videos as their primary study tool (evaluating). They chose these videos independently, consistently, and overwhelmingly. This reflected that faculty-created five-minute histology review videos fostered self-governing histology learning.

***The Self-Regulated Learning Theory-guided design worked — the 17-fold adoption rate reflected students fully embracing independent control of their histology learning.***

### Limitations

This study employed a within-subjects pre-post design in which students served as their own controls. The absence of a separate control group is a limitation. Randomization and a separate control group were not feasible. Splitting a simultaneously present student cohort into intervention and control groups within a live review laboratory session was not possible, as all students were entitled to equal access to the same review experience. Several factors suggest the observed score differences reflect more than methodological artefacts. Students first encountered these tissues as glass slides in face-to-face laboratory sessions. OlyVIA slides are digitized versions of the same glass slides, meaning students had multiple prior exposures to these images across different teaching modalities. The mock-OSPE images were OlyVIA virtual microscopy screenshots from the same digital slide library used in lectures, laboratory sessions, and videos. However, the mock-OSPE screenshots were captured at higher magnification to better visualize histological features, making direct image memorization from prior teaching sessions unlikely. The video was designed to teach histological identification criteria, not to familiarize students with specific images. It should also be noted that the mock-OSPE in this study was a formative teaching tool designed to reinforce histological identification criteria and expose knowledge gaps, not a summative assessment. A true OSPE is a face-to-face laboratory examination; the Google Forms quiz used here served a pedagogical rather than evaluative purpose. Despite this extensive prior exposure, pre-test scores remained low, suggesting that image memorization alone did not account for the post-test increase. The use of identical pre- and post-test questions introduces a potential test-retest effect; however, the brief interval between tests and the low pre-test scores suggest that familiarity with images and questions alone does not explain the observed score increases. The large effect size (Cohen’s d = 1.39) further supports the conclusion that the score difference exceeds what would be expected from question and image familiarity alone. The within-subjects design also offers methodological strengths: reduced inter-individual variability and a controlled comparison within the same session. Additionally, the use of personal devices and Google Forms provided instant score feedback for both pre- and post-test results, enabling self-assessment within the same session. Curriculum compression directly shaped the study design. The pre-post within-subjects design was the most practical approach within the constraints of a 35-minute scheduled laboratory session, while enabling video introduction and quantitative outcome measurement.

Pre-test and post-test mock-OSPE scores were analyzed at the question level rather than the individual student level due to the anonymous survey design, which prevented matching individual responses. While this approach reduced statistical power, the statistically significant results (*p* = 0.001, Cohen’s d = 1.39) point to substantial score increases despite this conservative approach. The mock-OSPE assessed blood vessel histology only — generalizability of performance advances to other organ systems was not directly measured.

Students who subsequently engaged in repeated viewing through spaced repetition may have shown even greater score increases. Future studies should examine whether repeated viewing is associated with greater score increases than single viewing alone, as progressive performance improvements were reported across cohorts with access to supplementary histology videos [48]. Students were not explicitly instructed on Spaced Repetition Theory principles. Future implementations could include brief guidance on spaced review strategies to optimize the use of brief on-demand videos.

Students self-reported consistently positive perceptions across three time points after experiencing the video series. Self-reported data cannot be independently verified. However, the positive perceptions align with documented Generation Z preferences for video-based learning [18].

The CVS Immediate survey questions on negative perceptions, in-class preferences, and out-of-class study methods were configured as single-select due to the author’s limited familiarity with Google Forms at the time of data collection. For these questions, statistical comparison was only possible between CVS post-final examination and Renal post-final examination time points. Three-time-point comparison was possible for positive perception features, which were correctly configured as multi-select throughout.

The longitudinal design followed the same student cohort across three time points within one semester but precluded assessment of long-term knowledge retention.

All materials and assessments were created and delivered by the author, minimizing variability and strengthening internal consistency of the intervention. This reflects a common methodological trade-off: controlling variability strengthens internal validity but may limit generalizability. The results could therefore partly reflect instructor-specific characteristics. Future studies should examine whether similar outcomes are observed with videos created by different instructors.

### Implications for medical education

Faculty-created five-minute histology review videos offer a pragmatic response to curriculum compression by extending education beyond limited classroom hours through 24/7 on-demand access. Brief videos are associated with significant performance advances and high voluntary adoption — findings consistent with substantial shifts in student learning behavior. The Generation Z learning profile aligns well with this approach — students valued hands-on laboratory learning and digital independent study as complementary rather than competing [18].

Videos should be released early enough to enable spaced repetition, with structured access windows that encourage distributed viewing rather than massing into last-minute study [53]. Each video should focus on a single learning objective and be integrated into the curriculum rather than offered as an optional supplement. Usage patterns and student performance should be monitored to refine content over time.

Faculty expertise remains essential in designing pedagogically sound microlearning resources aligned with curriculum objectives. Creating videos requires substantial faculty time investment — institutional recognition through workload allocation, promotion criteria, and technical resources would support broader adoption.

## Conclusions

Medical histology education faces a global crisis — curriculum compression has reduced histology laboratory hours while content expectations remain unchanged. Faculty-created five-minute histology review videos are an effective and reproducible microlearning component of a broader strategy to mitigate the effects of this curriculum compression. This study provides rigorous evidence across three Kirkpatrick levels, with findings marked by significant performance advances, high voluntary adoption, and a 17-fold behavioral shift in learning preferences. Theoretical frameworks served as interpretive lenses for contextualizing findings — not as verification or proof. Together, five theoretical frameworks offer a blueprint for effective histology microlearning: how to create effective content (Cognitive Load Theory, Multimedia Learning), how students engage with it (Spaced Repetition, Self-Regulated Learning Theory), and how to teach complex visual material (Variation Theory).

## Supplementary Information

Supplementary Material 1.

Supplementary Material 2.

Supplementary Material 3.

## Data Availability

Survey instruments are available upon request.

## Abbreviations

Mock-OSPE: Mock Objective Structured Practical Examination
CVS: Cardiovascular System
TBL: Team-Based Learning
SRL: Self-Regulated Learning
IRB: Institutional Review Board
AI: Artificial Intelligence
CLT: Cognitive Load Theory
