# Supplementary material for: Microlearning in medical histology: a longitudinal evaluation of faculty-created five-minute videos using five theoretical frameworks and Kirkpatrick assessment
Source: BMC Med Educ. 2026 Jul 14;26:1388. doi: 10.1186/s12909-026-09900-6 (PMC13520331; doi:10.1186/s12909-026-09900-6)
Supplement: Supplementary file 1 [file 12909_2026_9900_MOESM2_ESM.docx]

**Supplementary File S2. Data Collection Instruments**

**Part 1: Pre-Video mock-OSPE (12 questions)**

Your GPA (Highschool or UPP)

Your grade in Foundation Block

1 What is the structural name of this blood vessel

2 Give the name of the cells at the arrow

3 What is the structural name of this blood vessel at image B

4 What is the tissue at the arrow

5 What is the functional name of this blood vessel

6 How many layers of the smooth muscle can you see

7 What is the name of the layer at the arrow

8 What type of cells can you see in this layer

9 What is the structural name of this vessel

10 What is the key identification point

11 In which layer can you see this vessel at the arrow

12 What is the function of this vessel

**Part 2: Post-Video mock-OSPE and Immediate Survey**

Your GPA (Highschool or UPP)

Your grade in Foundation Block

1 What is the structural name of this blood vessel

2 Give the name of the cells at the arrow

3 What is the structural name of this blood vessel at image B

4 What is the tissue at the arrow

5 What is the functional name of this blood vessel

6 How many layers of the smooth muscle can you see

7 What is the name of the layer at the arrow

8 What type of cells can you see in this layer

9 What is the structural name of this vessel

10 What is the key identification point

11 In which layer can you see this vessel at the arrow

12 What is the function of this vessel

**Immediate Survey of part 2.**

1. Select the studying method you use the most. Select three you use the most.

☐ Reading histology textbook

☐ Reading PowerPoint lecture

☐ Listening to recorded PowerPoint lecture

☐ OlyVIA virtual microscopy

☐ Listen to faculty-created short videos

☐ Listen to YouTube videos

☐ Question bank

☐ Internet resources

________________________________________

2. Select the most meaningful teaching activity in the university. Select three you love the most.

☐ Histology lecture

☐ Histology face-to-face classical microscopy

☐ TBL (Team Based Learning)

☐ Peer teaching in evening labs

☐ Mock-OSPE

☐ Discussion with histology teacher

________________________________________

3. Did you find the 5-minute video summary about blood vessels helpful? If not, what are the reasons? Select 3 top Reasons.

☐ No, I prefer learning through reading

☐ No, I don’t typically study with videos

☐ No, I prefer interactive learning experiences rather than passive video watching

☐ No, the video lacked engaging or captivating elements

☐ No, the video was too fast-paced

☐ No, I prefer longer and more comprehensive explanations

☐ No, I was concerned that the information provided might be incomplete

☐ No, I was worried that it would not be sufficient for OSPE (Objective Structured Practical Examination)

☐ Other (please specify)

_______________________________________

4. Did you find the 5-minute video summary about blood vessels enjoyable? If yes, what were the reasons? Select 3 TOP Choices.

☐ Yes, I appreciate learning through listening rather than reading

☐ Yes, the video's concise length of 5 minutes minimized potential distractions

☐ Yes, the video presented complex concepts in a simplified and easy-to-understand manner

☐ Yes, the video effectively compared structures side-by-side for better understanding and retention

☐ Yes, hearing key medical terminologies in the video was beneficial

☐ Yes, the combination of seeing, reading, listening, and visual aids made the learning experience highly effective

☐ Yes, the video allowed me to skip reading a histology book, thus saving time

☐ Yes, the video provided an entertaining and educational experience simultaneously

☐ Yes, I can pause, rewind, and replay the video multiple times and access it conveniently anywhere

☐ Yes, the video utilized screenshots of the microscopic slides available in our histology lab

☐ Other (please specify)

**Part 3: CVS Post-Final Exam Survey**

1. How many times did you listen to the CVS videos?

☐ 0

☐ 1

☐ 2

☐ 3

☐ 4

☐ 5+

☐ Other (please specify)

________________________________________

2. If yes, which video did you listen to the most? Select all that apply.

☐ Talking heart (cardiac muscle and Purkinje fibers)

☐ Talking endocardium and epicardium

☐ Talking blood vessels

☐ Talking capillaries

☐ Talking veins

☐ Quiz of blood vessels

________________________________________

3. If yes, which video did you skip? Select all that apply

☐ Talking heart (cardiac muscle and Purkinje fibers)

☐ Talking endocardium and epicardium

☐ Talking blood vessels

☐ Talking capillaries

☐ Talking veins

☐ Quiz of blood vessels

________________________________________

4. I prefer the five summary videos.

☐ Yes

☐ No

☐ Other (please specify)

________________________________________

5. I prefer the one quiz-style video.

☐ Yes

☐ No

☐ Other (please specify)

________________________________________

6. Select the studying method you use the most. Select three you use the most.

☐ Reading histology textbook

☐ Reading PowerPoint lecture

☐ Listening to recorded PowerPoint lecture

☐ OlyVIA virtual microscopy

☐ Listen to faculty-created short videos

☐ Listen to YouTube videos

☐ Question bank

☐ Internet resources

________________________________________

7. Select the most meaningful teaching activity in the university. Select three you love the most.

☐ Histology lecture

☐ Histology face-to-face classical microscopy

☐ TBL (Team Based Learning)

☐ Peer teaching in evening labs

☐ Mock-OSPE

☐ Discussion with histology teacher

________________________________________

8. Did you find the 5-minute video summary about blood vessels helpful? If not, what are the reasons? Select 3 top Reasons.

☐ No, I prefer learning through reading

☐ No, I don’t typically study with videos

☐ No, I prefer interactive learning experiences rather than passive video watching

☐ No, the video lacked engaging or captivating elements

☐ No, the video was too fast-paced

☐ No, I prefer longer and more comprehensive explanations

☐ No, I was concerned that the information provided might be incomplete

☐ No, I was worried that it would not be sufficient for OSPE (Objective Structured Practical Examination)

☐ Other (please specify)

_______________________________________

9. Did you find the 5-minute video summary about blood vessels enjoyable? If yes, what were the reasons? Select 3 TOP Choices.

☐ Yes, I appreciate learning through listening rather than reading

☐ Yes, the video's concise length of 5 minutes minimized potential distractions

☐ Yes, the video presented complex concepts in a simplified and easy-to-understand manner

☐ Yes, the video effectively compared structures side-by-side for better understanding and retention

☐ Yes, hearing key medical terminologies in the video was beneficial

☐ Yes, the combination of seeing, reading, listening, and visual aids made the learning experience highly effective

☐ Yes, the video allowed me to skip reading a histology book, thus saving time

☐ Yes, the video provided an entertaining and educational experience simultaneously

☐ Yes, I can pause, rewind, and replay the video multiple times and access it conveniently anywhere

☐ Yes, the video utilized screenshots of the microscopic slides available in our histology lab

☐ Other (please specify)

10. During the three-week period when you had access to the videos, at what point(s) did you listen to them?

☐ Immediately after release

☐ The night before the exam

☐ During the exam period

☐ Throughout the three weeks

☐ Other (please specify)

**Part 4: Renal Post-Final Exam Survey**

1. How many times did you listen to the Renal videos?

☐ 0

☐ 1

☐ 2

☐ 3

☐ 4

☐ 5+

☐ Other (please specify)

________________________________________

2. If yes, which video did you listen to the most? Select all that apply.

☐ Talking kidney cortex

☐ Talking kidney: Bowman’s capsule and juxtaglomerular apparatus

☐ Talking kidney: papilla and junction

☐ Talking kidney: collecting ducts

☐ Talking kidney: blood flow, hormones, filtration

☐ Talking lower urinary system

________________________________________

3. If yes, which video did you skip? Select all that apply.

☐ Talking kidney cortex

☐ Talking kidney: Bowman’s capsule and juxtaglomerular apparatus

☐ Talking kidney: papilla and junction

☐ Talking kidney: collecting ducts

☐ Talking kidney: blood flow, hormones, filtration

☐ Talking lower urinary system

________________________________________

4. I prefer the six summary videos.

☐ Yes

☐ No

☐ Other (please specify)

________________________________________

5. I prefer quiz-style video.

☐ Yes

☐ No

☐ Other (please specify)

________________________________________

6. Select the studying method you use the most. Select three you use the most.

☐ Reading histology textbook

☐ Reading PowerPoint lecture

☐ Listening to recorded PowerPoint lecture

☐ OlyVIA virtual microscopy

☐ Listen to faculty-created short videos

☐ Listen to YouTube videos

☐ Question bank

☐ Internet resources

________________________________________

7. Select the most meaningful teaching activity in the university. Select three you love the most.

☐ Histology lecture

☐ Histology face-to-face classical microscopy

☐ TBL (Team-Based Learning)

☐ Peer teaching in evening labs

☐ Mock-OSPE

☐ Discussion with histology teacher

________________________________________

8. Did you find the 5-minute video summary about renal/ urinary system helpful? If not, what are the reasons? Select 3 top Reasons.

☐ No, I prefer learning through reading

☐ No, I don’t typically study with videos

☐ No, I prefer interactive learning experiences rather than passive video watching

☐ No, the video lacked engaging or captivating elements

☐ No, the video was too fast-paced

☐ No, I prefer longer and more comprehensive explanations

☐ No, I was concerned that the information provided might be incomplete

☐ No, I was worried that it would not be sufficient for OSPE (Objective Structured Practical Examination)

☐ Other (please specify)

_______________________________________

9. Did you find the 5-minute video summary about renal/ urinary system enjoyable? If yes, what were the reasons? SELECT 3 TOP Choices.

☐ Yes, I appreciate learning through listening rather than reading

☐ Yes, the video's concise length of 5 minutes minimized potential distractions

☐ Yes, the video presented complex concepts in a simplified and easy-to-understand manner

☐ Yes, the video effectively compared structures side-by-side for better understanding and retention

☐ Yes, hearing key medical terminologies in the video was beneficial

☐ Yes, the combination of seeing, reading, listening, and visual aids made the learning experience highly effective

☐ Yes, the video allowed me to skip reading a histology book, thus saving time

☐ Yes, the video provided an entertaining and educational experience simultaneously

☐ Yes, I can pause, rewind, and replay the video multiple times and access it conveniently anywhere

☐ Yes, the video utilized screenshots of the microscopic slides available in our histology lab

☐ Other (please specify)

10. During the one-week period when you had access to the videos, at what point(s) did you listen to them?

☐ Immediately after release

☐ The night before the exam

☐ During the exam period

☐ Throughout the entire week

☐ Other (please specify)

________________________________________

11. Additional comments

Please share any other thoughts you may have about the five-minute videos.

________________________________________

Thank you for taking the time to complete this survey.
